# The Fate of Coral Feeding Scars Following Predation by Individual Crown‐of‐Thorns Seastars

**DOI:** 10.1002/ece3.74285

**Published:** 2026-09-23

**Authors:** Shawna Andrea Foo, Matthew Clements, Adi Zweifler, Georgie Eggleton, Hayden Millican, Maria Byrne

**Affiliations:** ^1^ School of Life and Environmental Sciences The University of Sydney Sydney New South Wales Australia; ^2^ Marine Invertebrate Futures Group, School of Life and Environmental Sciences The University of Sydney Sydney New South Wales Australia

**Keywords:** *Acanthaster*, coral recovery, coral reef, corallivore, feeding rates, feeding scar, Great Barrier Reef, intermediate disturbance hypothesis

## Abstract

Aggregated crown‐of‐thorns seastars (*Acanthaster* spp., COTS) in outbreak populations create large feeding scars that often result in complete coral colony mortality. In contrast, feeding scars produced by individual COTS may retain living coral tissue remnants, but the responses of these remnants post predation remain unknown. We tracked the fate of discrete feeding scars left after predation by individual *Acanthaster* cf. *solaris* from a low‐density population at One Tree Reef on the Great Barrier Reef. We found that the average feeding scar size did not differ by season, despite variation in coral genera and colony sizes preyed on. Most scars (72%) were left with remnants around their perimeter. The scars were initially colonised by turf algae, with some crustose coralline algae (CCA) present by 10 weeks post‐predation. By 22 weeks, 26% of scars showed signs of regenerating coral tissue. Regeneration occurred predominantly in *Montipora*. In contrast, *Acropora* scars remained covered by turf or CCA. COTS appear to consume a relatively consistent amount of coral tissue per feeding event; consequently, remnant extent is primarily determined by colony size and whether a colony exceeds the typical amount of tissue consumed during a single feeding event. Thus, coral survival after COTS predation appears to be governed by two interacting processes: the likelihood that a remnant is left following predation and the regenerative capacity of that remnant. These findings demonstrate that predation by low‐density COTS does not necessarily result in coral colony mortality and highlights sublethal predation and live tissue survival as important components of coral persistence and recovery. This may help maintain coral community diversity by limiting fast‐growing taxa, consistent with the intermediate disturbance hypothesis, and is an important consideration in COTS management.

## Graphical abstract

Low density populations of crown‐of‐thorns seastars (COTS) create consistent‐sized feeding scars across diverse coral taxa at One Tree Reef on the southern Great Barrier Reef. While most feeding scars were initially colonised by turf and crustose coralline algae, a quarter showed signs of live coral tissue regeneration after 22 weeks, with *Montipora* regenerating more than *Acropora*. These results suggest that low‐density COTS predation can help to moderate fast‐growing taxa and support coral community recovery, aligning with the intermediate disturbance hypothesis.

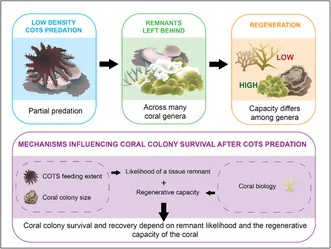

## 1 Introduction

Crown‐of‐thorns seastars (COTS, *Acanthaster* spp.) are a major cause of coral mortality in Indo‐Pacific reefs, especially in high‐density outbreak populations (Pratchett et al. 2021; Foo et al. 2024). Most studies of these seastars have focussed on the western Pacific species, *Acanthaster* cf. *solaris* in ‘boom and bust’ outbreak populations (Foo et al. 2024; Uthicke et al. 2023; Byrne and Wolfe 2027). In these populations, COTS form feeding aggregations that create large feeding scars on coral colonies denuded of live tissue (Pratchett et al. 2014). As they move in search of prey, they leave dead coral in their wake. There are fewer studies of COTS in stable populations (Millican et al. 2024). In these low‐density populations, COTS are less motile (Keesing 1990), are local residents on reefs, show homing behaviour and the feeding scars they create are smaller as they are typically created by a single seastar (Millican et al. 2024; Ling et al. 2020).

The bare skeleton of the feeding scars created by COTS predation on corals has been observed to be rapidly overgrown by algae post‐predation (Chesher 1969; Larkum 1988). In Guam, algal colonisation of the skeletons of *Acropora* preyed on by COTS began within 24 h, with blue‐green algae initially dominant and brown algae prevailing after 29 days (Belk and Belk 1975). Similar patterns were observed on the Great Barrier Reef (GBR), where increased algal cover on feeding scars created by COTS enhanced nitrogen fixation, which peaked 3–6 months post‐predation and declined by 9 months (Larkum 1988). The contribution of this to the total nitrogen‐fixation budget was small but could be considerable for outbreaking populations (Larkum 1988).

After predation by COTS, live coral can remain as tissue left on partially consumed colonies and tissue that was physically inaccessible to the seastars (Branham 1973; Randall 1973; Colin 1977; Done 1985; Colgan 1982). These surviving parts of the coral colony have been referred to as “remnants” (Randall 1973). It has been hypothesised that remnants can regenerate to increase coral cover (Colgan 1982) although this has not been investigated through tracking of individual feeding scars. Done (1985) counted the number of remnants (patches of living coral) per coral genus through systematic searching of transects and quadrats every 6 months for 1.5 years after a COTS outbreak on the GBR. Remnants remaining after predation were present at high densities (20–60 per m^2^) across a range of coral genera, with high viability over a year post COTS predation (Done 1985). Most of the remnants (44%) were *Porites*, with *Acropora* having minimal remnants (Done 1985). In *Porites*, surviving remnants that were present in isolated patches across originally large coral colonies separated to form subcolonies (DeVantier and Done 2007). It was suggested that over years to decades, these patches may fuse due to tissue regrowth over the intervening dead coral surface (DeVantier and Done 2007), although this has not been investigated.

As a clonal animal, the ability to regenerate lost or injured tissue in corals is critical to their survival. Studies have found that corals with large wounds and small perimeters of remaining live tissue regenerate less well, especially in food limited scenarios (Henry and Hart 2005). The ability of a coral to regenerate is positively correlated with increasing colony size (Kramarsky‐Winter and Loya 2000). If remnant live coral remaining after predation is substantial, regeneration of coral tissue may contribute to re‐establishment of coral cover post COTS predation (Done 1985). Tracking feeding scars and the regenerative capacity of remnants over denuded scar skeleton is especially important for reefs in the context of stable COTS populations. In such reefs, COTS predation may act as a long‐term press stressor. Evidence of homing behaviour in non‐outbreaking COTS (Millican et al. 2024; Ling et al. 2020) raises the question of whether a balance between predator and prey can be maintained over time to support resident low‐density COTS populations.

As per the “Intermediate Disturbance Hypothesis” (Connell 1979), the feeding behaviour of COTS may contribute to the balance between competition and colonisation on coral reefs, leading to increases in species diversity due to the reduction of fast‐growing species (Colgan 1987; Done et al. 1997). In Japan, populations of *A*. cf. *solaris* prevented fast‐growing *Acropora* from dominating the reef, facilitating the growth of slower growing coral species (Keesing 1993). Previous work found that 12 years after a large COTS outbreak, coral species cover and richness exceeded measurements prior to the outbreak (Colgan 1987). Because the underlying structural integrity of the skeleton remained intact after COTS predation, this facilitated reef recovery as recruits utilised the stable substrate for larval settlement (Colgan 1987). While not related to COTS, large *Porites* colonies which had severe partial mortality after bleaching showed regrowth of tissue over the denuded coral skeleton from cryptic remnants (Roff et al. 2014). Therefore, in areas where COTS occur in stable, low‐density populations, predation on their preferred prey, *Acropora*, may facilitate establishment of non‐preferred species as per the intermediate disturbance hypothesis.

We investigated the feeding scars created by COTS from low‐density populations of *Acanthaster* cf. *solaris* at One Tree Reef (OTR), southern GBR. This population and associated corals have persisted for decades (Souter et al. 1997). Feeding scars created by COTS from stable populations tend to be discrete, making their fate possible to track over time. Due to their homing behaviour, feeding scars can be attributed to individual COTS (Millican et al. 2024). Thus, for the first time, we followed the fate of discrete scars created by individual seastars over 22 weeks, where scars were visited across multiple timepoints to observe their benthic succession. We assessed the seasonal and coral genus‐specific differences in COTS feeding scars and factors that impacted the probability of remnant occurrence. Where remnant coral tissue remained, we assessed the importance of this tissue following predation, with respect to potential for regenerative re‐sheeting of the denuded skeleton as described post bleaching (Roff et al. 2014). These data were used to investigate how COTS predation aligns with the intermediate disturbance hypothesis at our study site.

## 2 Methods

### 2.1 Species and Sites

Although the taxonomy of the west Pacific species of COTS that occurs on the GBR remains to be determined due to lack of type specimen and a reliable type locality (Haszprunar and Spies 2014; Hutchings et al. 2025), we apply the name in current use, *Acanthaste*r cf. *solaris*. Crown‐of‐thorns seastar feeding scar data were collected at One Tree Reef (23°30′ S, 152°6′ E) from two sites: Shark Alley, First Lagoon (23°30′ 28.344″ S, 152° 5′ 23.309″ E) and Second Lagoon (23° 30′ 19.524″ S, 152° 3′ 28.403″ E) between 2022 and 2024. For each analysis, the dataset used is specified in the respective sections. The COTS at these two sites are large and similar in size (mean disc diameter = 37–38 cm) and recent population counts indicate a density of ~8 per ha in Shark Alley and ~18 per ha in Second Lagoon (Millican et al. 2024).

Previous studies show that the percentage of live coral cover does not significantly differ between Shark Alley and Second Lagoon sites (40% and 44%, respectively (Millican et al. 2024)). The dominant genera contributing to coral cover is primarily *Porites* and *Isopora* in Shark Alley and *Acropora*, *Porites* and *Montipora* in Second Lagoon (Millican et al. 2024).

### 2.2 Feeding Scar Size Across Seasons and Coral Genera

To assess feeding scar size, we investigated scars created by the population of COTS in Shark Alley. Only feeding scars from Shark Alley were used for this analysis due to site access across time, tides and sea conditions.

We photographed every scar that was encountered and accessible for photography across winter (June 2022, May 2023, *n* = 46 and 109 respectively), summer (February 2023, *n* = 24) and spring (October 2023, *n* = 92). All scars encountered (total *n* = 271) were photographed regardless of coral genus or scar size to obtain a random and representative sample of the corals preyed on by the resident COTS. Feeding scars were photographed from the angle that captured the greatest 2D surface area using underwater cameras (Olympus Tough TG‐6) alongside a ruler next to the coral for scale. Only fresh feeding scars devoid of live coral tissue with bare skeleton exposed were photographed. To identify coral genera, multiple images of each colony were taken, including close‐ups of corallites, and identification was guided by a coral identification guide (Kelley 2022).

From each photograph, the 2D feeding scar surface area (cm^2^) was calculated in ImageJ (Rasband 2018) by outlining the feeding scar perimeter. As the OTI population of COTS exhibits homing behaviour, feeding scars represent discrete events attributable to individual COTS (Millican et al. 2024). To investigate mean feeding scar size, we used bootstrapping (10,000 iterations) to generate 95% confidence intervals (CIs) across months and coral genera. Non‐overlapping CIs were interpreted as evidence of significant differences in mean scar size (Smith 1997; Cumming and Finch 2005). To ensure sufficient replication for the genus‐level comparison, the smallest colony sized branching genera (*Seriatopora* and *Pocillopora*) were combined into a single group. Coral genera with low sample sizes (*n* < 4) were excluded from this analysis.

### 2.3 Factors Affecting Feeding Scars and Remnants

To investigate relationships between feeding scar characteristics, remnants, coral genus and colony size, we used data from scars created by COTS in Shark Alley and Second Lagoon in May 2023. Restricting analyses to a single sampling period ensured that scar age was consistent across all observations, allowing data from both sites to be compared (*n* = 260; Second Lagoon = 151; Shark Alley = 109). Analyses involving coral genus were restricted to genera represented by at least five observations (*Acropora*, *Porites*, *Seriatopora*, *Isopora*, *Montipora* and *Pocillopora*).

For each feeding scar, we recorded whether any live coral tissue remained following predation (remnant present) or whether the colony had been completely consumed (fully eaten, Table 1). Where possible, alongside the 2D feeding scar surface area measurement, the 2D area of the entire coral colony (cm^2^) was measured in ImageJ (Rasband 2018), allowing calculation of the proportion of the colony consumed (feeding scar area/colony area; *n* = 104 of 260, Table 2).

**TABLE 1:** COTS feeding outcomes by coral genus and site.

Genus	Second Lagoon				Shark Alley			
	*n*	Remnants present	Fully eaten	Remnant rate (%)	*n*	Remnants present	Fully eaten	Remnant rate (%)
*Acropora*	65	62	3	95.4	15	7	8	46.7
*Cyphastrea*	1	1	0	100	1	0	1	0
*Favites*	—	—	—	—	1	0	1	0
*Goniopora*	—	—	—	—	1	1	0	100
*Isopora*	9	7	2	77.8	21	15	6	71.4
*Montipora*	18	17	1	94.4	6	4	2	66.7
*Platygyra*	—	—	—	—	1	1	0	100
*Pocillopora*	13	4	9	30.8	7	2	5	28.6
*Porites*	15	12	3	80	40	32	8	80
*Seriatopora*	30	17	13	56.7	15	5	10	33.3
*Turbinaria*	—	—	—	—	1	1	0	100
Total	151	120	31	80	109	68	41	62

*Note:* For each scar, we recorded whether any live coral tissue remained following the feeding event (remnants present), or whether the colony had been entirely consumed (fully eaten) with the rate (%) calculated showing the percent of corals with remnants left behind after feeding, per total number of corals across genera and site. The dash indicates no data available for the respective genus.

**TABLE 2:** Feeding scar versus total coral colony size.

Genus	*n*	Mean colony size (cm^2^) ± SD	Mean scar size (cm^2^) ± SD	Mean % eaten	Coral colonies with remnants (%)
Second Lagoon (*n* = 51)					
*Acropora*	7	85.9 ± 66.9	47 ± 35.6	69.4	71
*Isopora*	4	90.4 ± 90	74.6 ± 70.6	86.1	50
*Montipora*	3	710.6 ± 710	278.2 ± 204.1	61.6	67
*Pocillopora*	12	69.9 ± 52.9	68.3 ± 49.5	97.4	25
*Porites*	4	277.2 ± 352.3	123.3 ± 120	71.2	75
*Seriatopora*	21	74.5 ± 78.4	64.2 ± 81.6	82.2	48
Shark Alley (*n* = 55)					
*Acropora*	7	43.8 ± 31.7	43.4 ± 31.6	99.1	14
*Isopora*	10	150.8 ± 228.4	126.7 ± 193.5	87.6	60
*Montipora*	5	527.5 ± 977.5	270.5 ± 419.2	78.9	60
*Pocillopora*	7	41 ± 28.8	36.1 ± 28	90.2	29
*Porites*	10	66.9 ± 69.2	52.1 ± 47.5	82.5	70
*Seriatopora*	14	49.4 ± 34.6	47.7 ± 33.4	94.9	29

*Note:* The mean colony and feeding scar size per genus are shown, where the Mean % eaten = scar area ÷ colony area × 100. The percentage of coral colonies that were left with tissue remnants is shown for the subset of coral where both feeding scar and whole colony size could be measured (*n* = 104).

To investigate factors influencing remnant occurrence, binomial logistic regression models were fitted with feeding outcome (remnant present = 1; fully eaten = 0) as the response variable. We examined whether coral genus and site influenced remnant occurrence, and, where colony area was available, coral genus, site and log‐transformed colony area. Colony area was log‐transformed to reduce right‐skewness and because the relationship between colony size and the response was expected to be proportional rather than linear. Models were compared using likelihood ratio tests and Nagelkerke's pseudo‐*R* ^2^.

To investigate feeding events in which a high proportion of the colony was consumed, feeding events in which remnants remained despite ≥ 80% of the colony area having been consumed were identified and characterised by coral genus, site, colony area and remnant area to determine whether they occurred across a range of colony sizes.

To examine whether feeding scar area scaled proportionally with colony area, a linear regression of log‐transformed feeding scar area against log‐transformed colony area was fitted, including coral genus and site as covariates. The regression slope was compared with 1 using its 95% confidence interval, and site‐specific slopes were estimated to assess consistency between locations.

All analyses were conducted in R (v4.4.2).

### 2.4 Tracking Individual Feeding Scars Over Time

**FIGURE 1:**
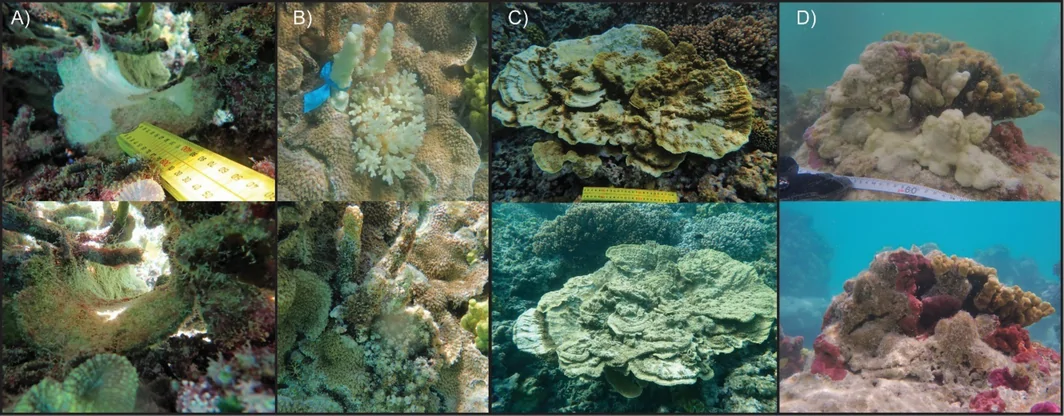
Post‐predation changes to the surface of crown‐of‐thorns seastar feeding scars over time. The top panels show the bare scar at time zero. The bottom panels show examples of the four categories: Turf (A), crustose coralline algae (B), regenerated coral tissue (C) and mixed assemblage (D), in this case regenerated coral tissue and turf. Further, detailed photographs of individual scar tracking and close‐ups of the different categories are shown in Figure 2.

**FIGURE 2:**
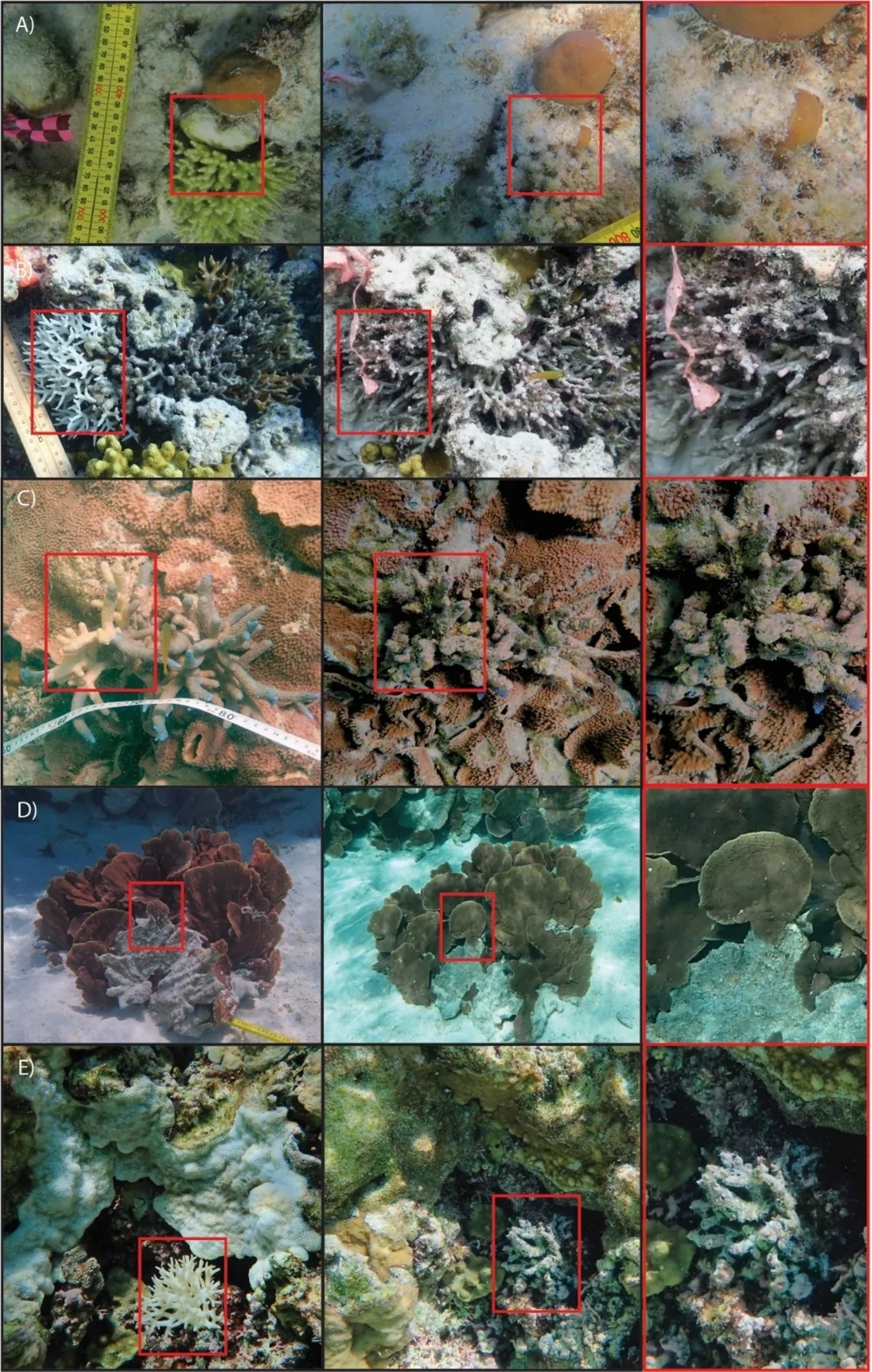
Detailed images of individual feeding scars showing the changes to the surface over time. The left panels display the fresh scar, the middle panels display the same scar after 22 weeks and the right panels display a close‐up of the area indicated by the red box. Panel A shows a *Porites* scar with growth of remnants and a *Seriatopora* scar that remained turfed throughout the observation period. Panel B shows a *Seriatopora* scar colonised by crustose coralline algae (CCA); although remnants were left behind, no regenerative growth occurred. Panel C shows an *Acropora* scar largely colonised by CCA and some turf algae. Despite a large remnant initially remaining, the entire colony was eventually overgrown. Panel D shows a scar on a *Montipora* colony with regrowth from a remnant. Panel E displays a *Porites* and *Seriatopora* scar covered by a dense CCA community.

To track the fate of COTS feeding scars, a total of 71 unique feeding scars were identified for monitoring in Shark Alley (*n* = 52) and in Second Lagoon (*n* = 19) in November 2023. A subset of these scars was rephotographed after 1 (*n* = 25), 10 (*n* = 22) and 22 (*n* = 37) weeks post‐predation subject to relocation (dependent on sea conditions). The scars were relocated through using a combination of GPS coordinates, marker buoys, flagging tape and identification of landmarks on the reef. During each visit, the colonisation of the surface of the scar skeleton was classified into four categories (Figures 1 and 2): (1) Turf: single‐ to multi‐species assemblages of benthic algae, (2) Crustose coralline algae (CCA): encrusting, calcium carbonate depositing red algae, (3) Coral: regenerated coral tissue over the originally bare denuded feeding scar, and (4) Mixed: a combination of 1–3.

### 2.5 2D Versus 3D Measurements of Feeding Scars

We trialled the use of three‐ (3D) and two‐ (2D) dimensional imaging of the feeding scars to determine if 2D measurements would provide sufficient information on scar size. A comparison of 2D versus 3D measurements of feeding scars showed that while 3D measurements estimate a significantly larger mean feeding scar size than 2D measurements, this did not differ across complex (branching) or simple (encrusting, solitary, massive) type corals and so for the purpose of our investigation with respect to our focal aims and fate of scars, 2D measurements were used for further comparisons. Unless otherwise stated, all references to area refer to 2D measurements. Due to greater efficiency, 2D measurements allowed us to assess and track a high number of feeding scars across a broad range of coral types, avoiding the logistical challenges of 3D photogrammetry, such as extensive photo collection, lighting constraints and processing time (House et al. 2018). Further methods and results for these analyses are in Supporting Information Methods, Figure S1 and Tables S1 and S2.

## 3 Results

### 3.1 Feeding Scar Size Across Time and Genera

**FIGURE 3:**
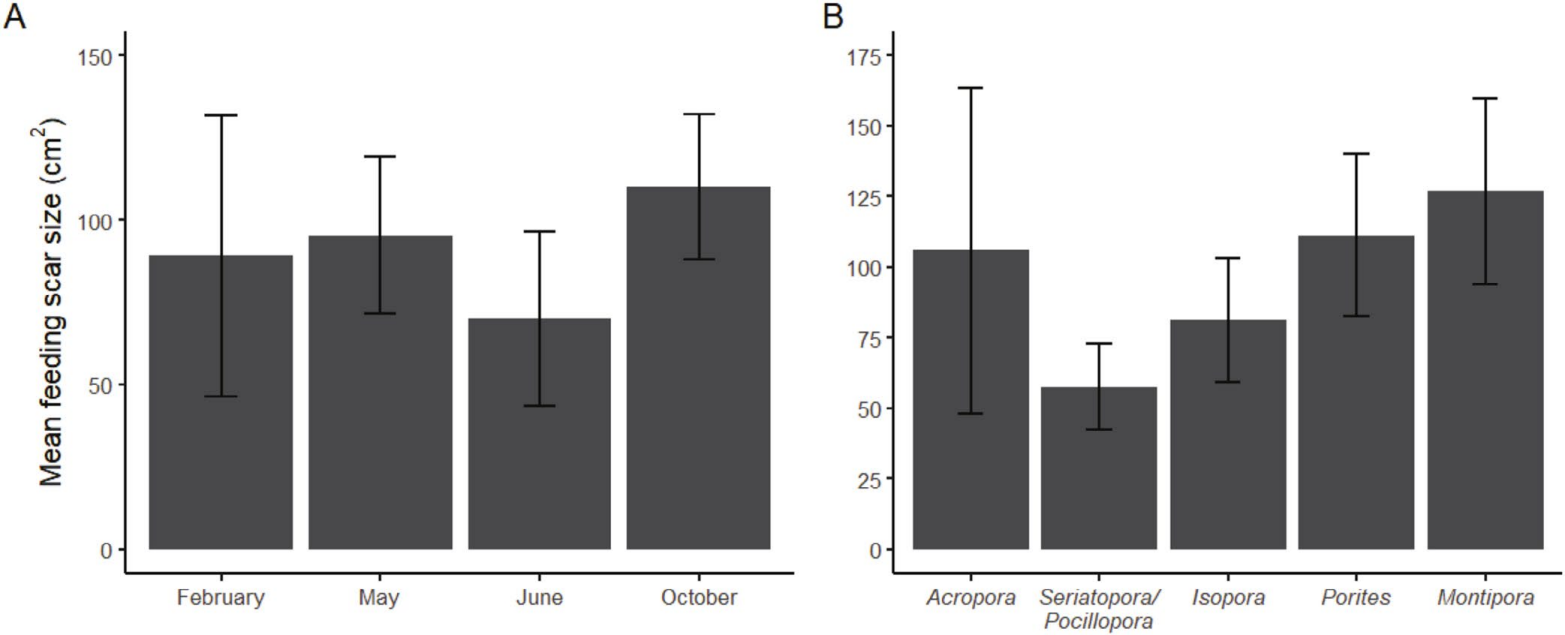
Mean individual feeding scar size across (A) seasons and (B) coral genera. Seasons were defined as winter (May–June), spring (October) and summer (February). The figures show mean feeding scar size and 95% confidence intervals. *N* = 24, 109, 46, 92 for February, May, June and October respectively. *N* = 28, 37, 80, 32, 85 for *Acropora*, *Seriatopora/Pocillopora*, *Isopora*, *Porites* and *Montipora* respectively.

The 2D surface area of the 271 scars photographed in Shark Alley ranged from 1.44 to 760.29 cm^2^ (overall mean ± SE: 95.46 ± 6.78 cm^2^). Bootstrap analysis showed that the mean area of individual feeding scars did not differ significantly across seasons (Figure 3A). Across coral genera, the mean size of the feeding scars created by individual COTS on *Seriatopora/Pocillopora* corals was significantly smaller than those on *Porites* and *Montipora* (Figure 3B), while scar sizes did not differ significantly among other genera.

### 3.2 Factors Affecting Feeding Scar Size and Coral Tissue Remnants

Considering the dataset from May 2023, across both sites, remnants were left after COTS predation in 188 of 260 feeding scars (72%), with 72 cases of complete colony consumption. We found that both genus (*p* < 0.001) and site (*p* < 0.001) were strong predictors of whether COTS left a remnant (Table S3). There was a higher likelihood of a remnant being left in Second Lagoon (80% of feeding events) compared with 62% at Shark Alley (Table 1).

Post hoc pairwise comparisons between genera showed significant differences in remnant probability across several genus pairs. *Acropora* had a significantly higher remnant probability than both *Pocillopora* (*p* < 0.0001; predicted remnant probabilities: 0.86 vs. 0.30) and *Seriatopora* (*p* < 0.0001; 0.86 vs. 0.49) and, similarly for *Porites* in comparison to *Pocillopora* (*p* = 0.001; 0.80 vs. 0.30) and *Seriatopora* (*p* = 0.004; 0.80 vs. 0.49). *Montipora* also had significantly higher remnants compared to *Pocillopora* (*p* = 0.001; 0.88 vs. 0.30) and *Seriatopora* (*p* = 0.008; 0.88 vs. 0.49). *Isopora* had significantly more remnants than *Pocillopora* (*p* = 0.008; 0.73 vs. 0.30).

With respect to the size of the coral colonies preyed on by COTS, measurements of the surface area of the total coral colony show the large size range of the colonies preyed on (Table 2). The mean surface area of *Pocillopora* colonies was the smallest (mean ± SD; 41 ± 28.8 cm^2^), whereas *Montipora* colonies were the largest colonies preyed on (mean ± SD; 710.6 ± 710 cm^2^). Across all genera, COTS consumed a large proportion of the colony (> 60%; Table 2).

Where colony area data were available, the logistic regression model found that genus and site were not significant predictors of whether COTS left a remnant (Table S4); however, colony size was significant (*p* = 0.004). The odds ratio for log colony area was 2.13 (95% CI: 1.32–3.76), indicating that larger colonies were more likely to have a remnant left. This means that for each unit increase in log colony area, the odds of a remnant roughly double.

**FIGURE 4:**
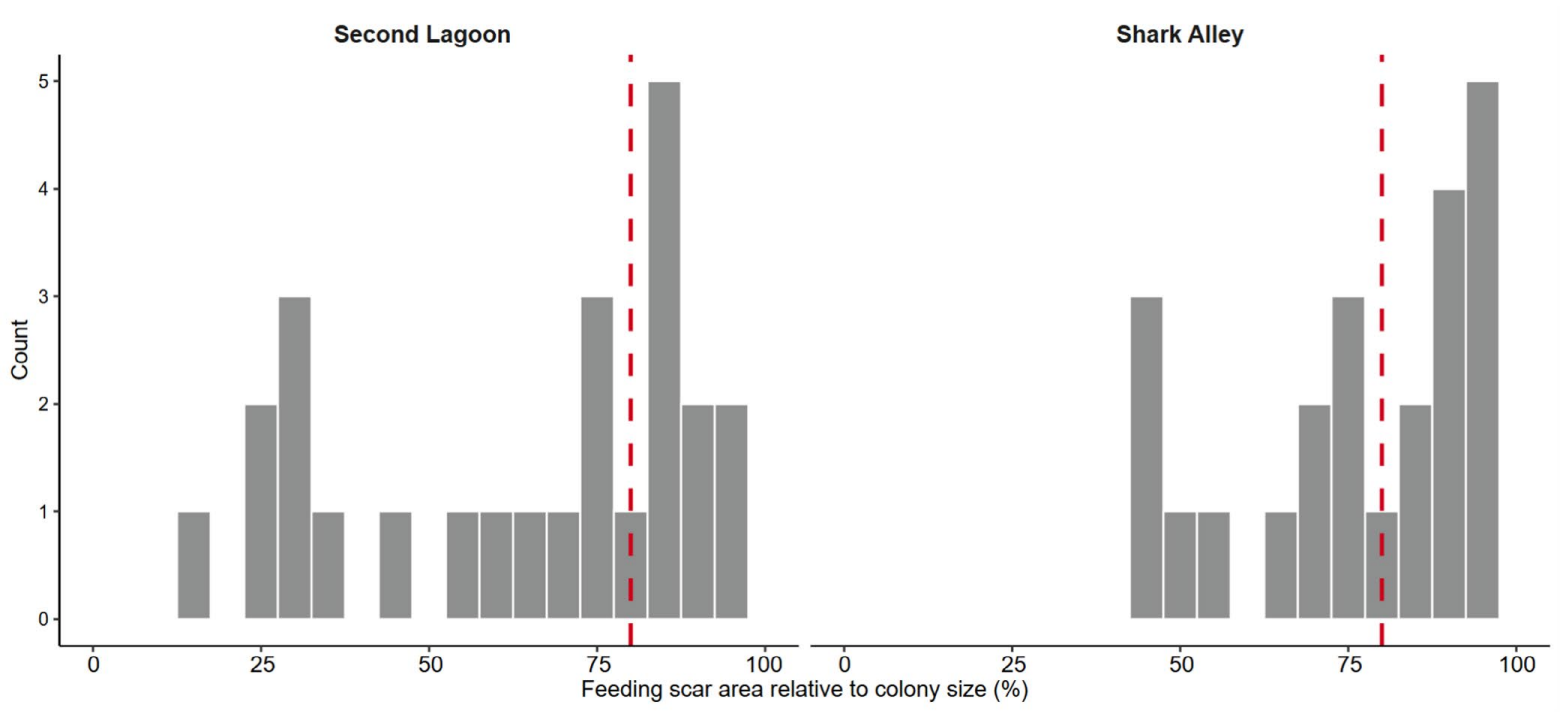
Distribution of the proportion of coral colony area consumed (%) for feeding events in which a remnant remained in the colonies at Second Lagoon (*n* = 25) and Shark Alley (*n* = 23). The dashed red line marks the 80% threshold used to identify cases in which live tissue remained despite extensive (> 80%) colony consumption.

Of the events where a remnant was left, the percentage of the colony consumed covered a broad range (Figure 4). 20 cases (41%) had a feeding scar which represented more than 80% of the colony: nine at Second Lagoon (36%) and eleven at Shark Alley (48%). Further exploring only these cases of > 80% colony consumption, we found that events spanned six genera with colony sizes ranging from 25.8 cm^2^ to 624.8 cm^2^ (Table S5).

**FIGURE 5:**
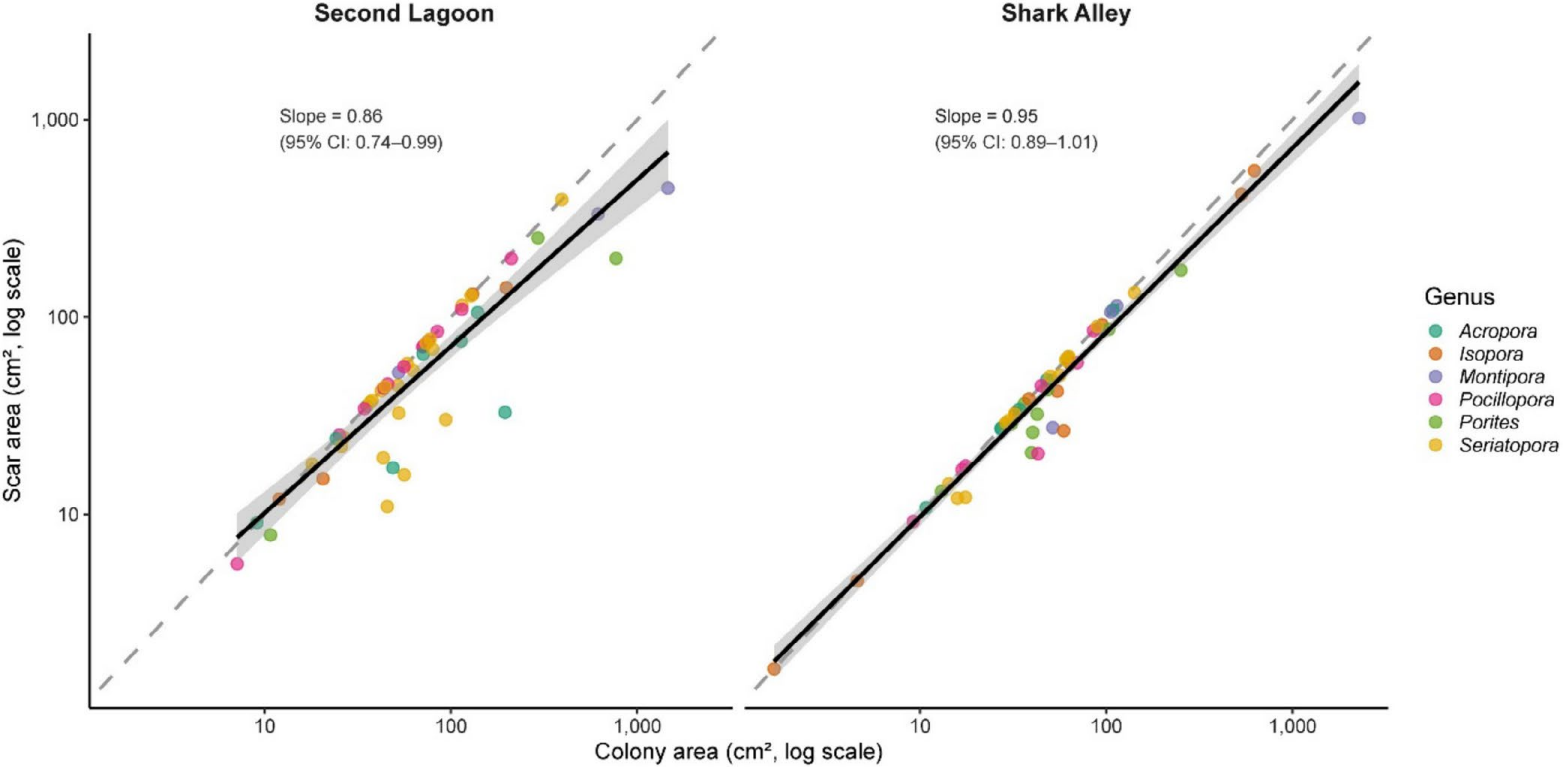
Feeding scar area (cm^2^) plotted against coral colony area (cm^2^) on log–log axes for colonies in Second Lagoon and Shark Alley. Each point represents one feeding scar event, coloured by coral genus. The dashed line indicates the 1:1 relationship where scar area is proportional to colony area (i.e., the colony was fully consumed). The solid line is an ordinary least squares regression fit (controlling for genus) with 95% confidence interval (shaded). A slope significantly less than 1 indicates that the feeding scar area is not proportional to colony area.

To examine whether feeding scar area scaled proportionally with colony size, site‐specific relationships between feeding scar area and colony area were assessed (Figure 5). At Second Lagoon, feeding scar area increased significantly less than proportionally with colony area (slope = 0.86, 95% CI = 0.74–0.99), indicating that larger colonies tended to retain a greater proportion of living tissue following a feeding event. In contrast, at Shark Alley, feeding scar area increased approximately in proportion to colony area (slope = 0.95, 95% CI = 0.89–1.01), with the slope not differing significantly from one.

### 3.3 Feeding Scar Fate

**FIGURE 6:**
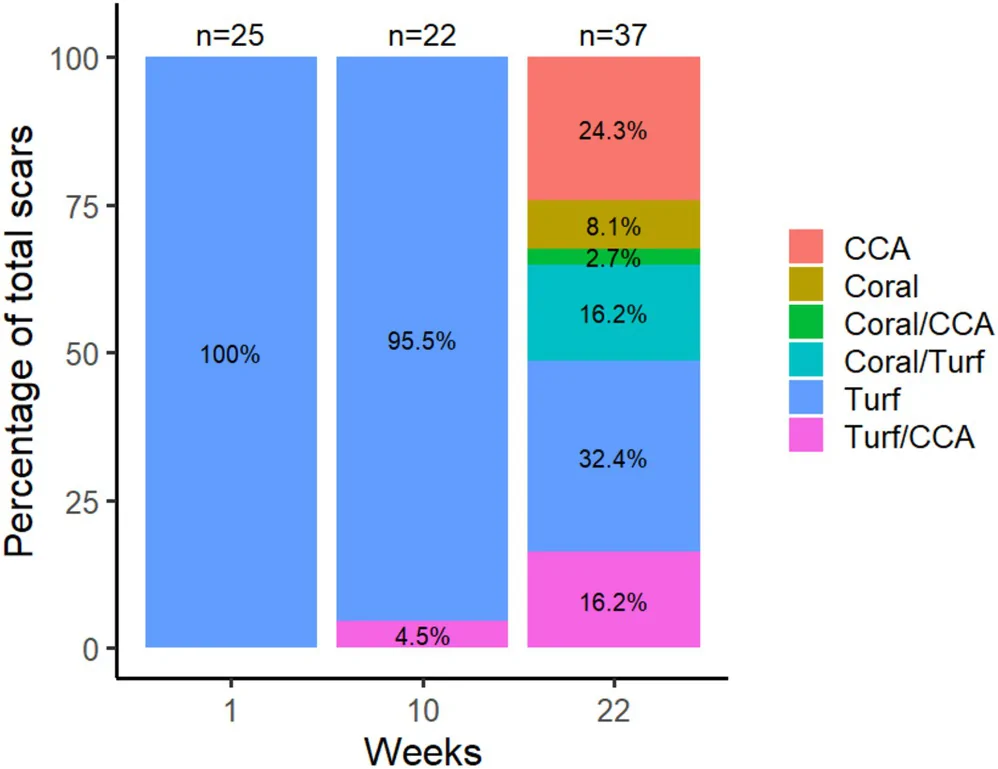
Temporal changes in the appearance of COTS feeding scars following predation. Feeding scars were initially photographed when fresh and a subset was re‐photographed 1, 10 and 22 weeks after predation. Bars show the percentage of feeding scars classified as turf, crustose coralline algae (CCA), coral (regenerated coral tissue covering the scar surface), or a combination of these categories at each time point. Of the 71 feeding scars initially identified, 25 were re‐photographed after 1 week, 22 after 10 weeks and 37 after 22 weeks (displayed above corresponding bars).

Changes in the surface of the 71 feeding scars tracked over time is shown in Figure 6. In the early weeks after initial COTS predation (weeks 1 and 10), most feeding scars were colonised by turf algae. A small proportion showed early signs of colonisation by crustose coralline algae (CCA) after 10 weeks (Figure 6). By week 22, scar surfaces exhibited a diverse range of cover types, with 48.6% of scars covered by turf algae or CCA, or a mix of both (Figures 1, 2 and 6). Notably, regenerated coral tissue over the scar surface was observed at this time point. All feeding scars tracked retained their skeletal structure over the 22 weeks of this study.

**FIGURE 7:**
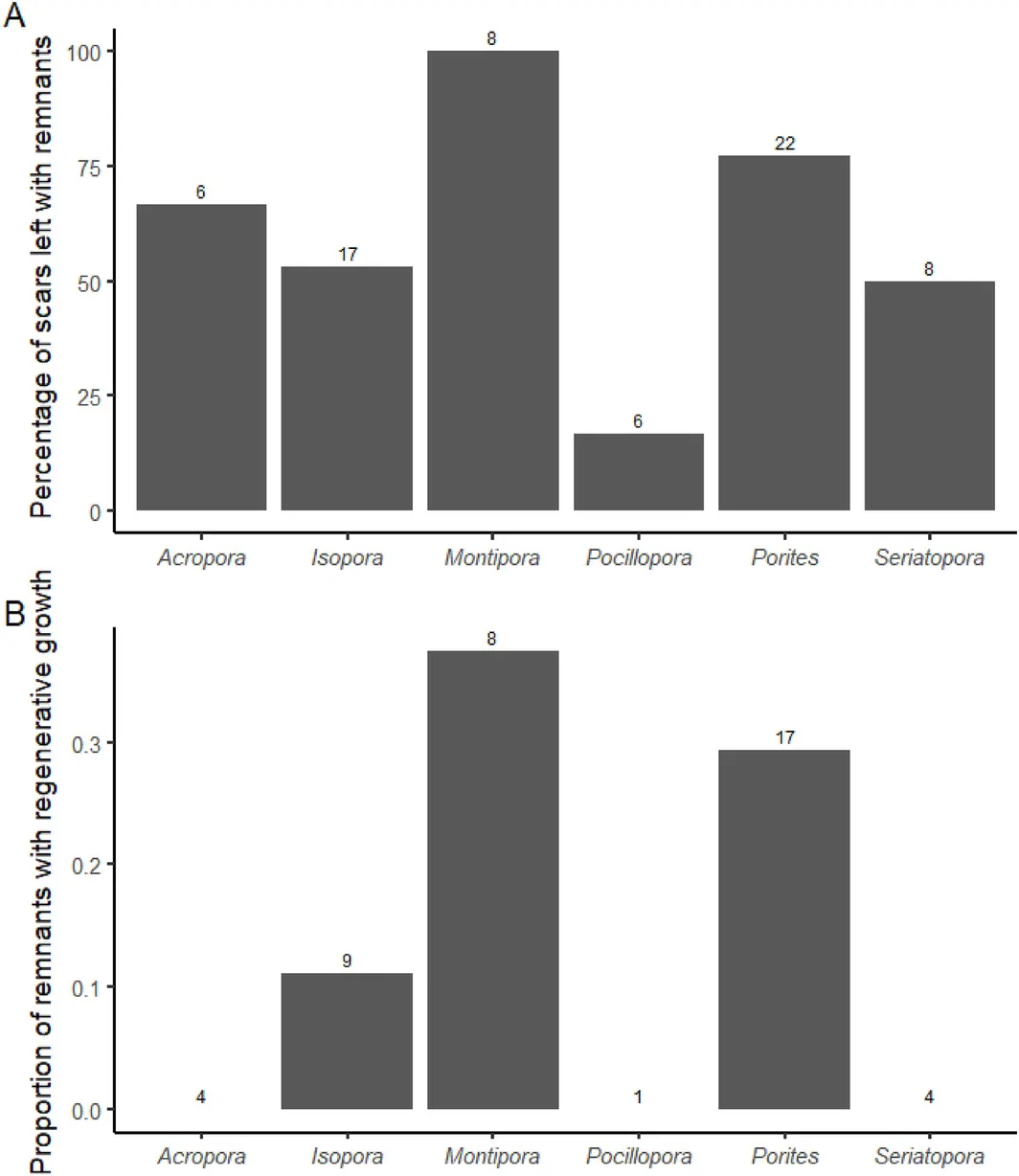
Proportion of tracked feeding scars with coral remnants and subsequent regenerative growth, by coral genus. (A) Proportion of feeding scars with live coral remnants immediately following COTS predation (time 0). (B) Proportion of feeding scars with remnants that showed regenerative growth by 22 weeks post‐predation. Sample sizes for each coral genus are shown above the corresponding bars; in panel (B), proportions are calculated only from feeding scars in which remnants were present.

Of the 71 individual feeding scars followed in the tracking study, 46 of these had living coral tissue around the outside edges of the scar (remnants). The percentage of corals with remnants left behind differed across the coral genera, where for all *Montipora* colonies observed, remnants were left behind (Figure 7A). The 25 colonies that were completely denuded of tissue showed no evidence of regeneration, indicating that there was no source of recovery for these corals. For the 46 scars that had living tissue around the edge, 74% showed no regeneration, while the remaining 26% exhibited regenerative growth over at least part of the feeding scar by the final timepoint at 22 weeks. Regenerative growth was only observed for *Isopora, Montipora* and *Porites*, and not for the branching genera (Figure 7B).

## 4 Discussion

The low‐density nature of the OTR population of COTS with their formation of discrete feeding scars allowed an investigation of individual scars and tracking the fate of individual feeding scars over time. While monitoring scars, it was observed that most (72%) corals experienced partial predation, where live coral tissue on the colony (remnants) was left behind. This was influenced by coral genus and colony size. Over time, some feeding scars (26%) showed evidence of coral tissue regeneration over the denuded skeleton.

### 4.1 Crown‐of‐Thorns Feeding Scar Size Is Similar Across Seasons

Our results show that feeding scar size, the area consumed by *Acanthaster* cf. *solaris* on a given coral colony, was similar across seasons at our study site. We found that feeding scar size was similar across most coral genera, with the exception of *Seriatopora* and *Pocillopora*, where feeding scars were significantly smaller compared with other genera. COTS appears to eat a similar amount of tissue per feeding event throughout the year at OTR, suggesting a relatively consistent feeding pressure that allows for live coral tissue remnants to be left behind.

### 4.2 Remnants Are Common and Differ Among Coral Genera but Is Primarily Driven by Colony Size

A substantial proportion of feeding events left viable coral tissue remnants behind (72%). The probability of remnants was higher in Second Lagoon than Shark Alley, with a lower probability of remnants for *Seriatopora* and *Pocillopora* compared to the other genera. A previous study reported relatively high numbers of remnants in *Porites* and *Montipora* (Done 1985), consistent with our findings. Notably, in our study, remnants were present in a relatively high number of *Acropora*, *Pocillopora* and *Seriatopora* colonies, an interesting finding given that previously, remnants have been found to be inconsequential for *Acropora* (Done 1985; DeVantier and Done 2007).

Across all coral genera, COTS typically consumed a large proportion of each colony (> 60%), such that any remaining live tissue represented only a small fraction of the original colony. Although remnant probability initially appeared to differ among genera, colony size emerged as the strongest predictor once incorporated into the model, explaining more variation than either site or genus. Larger colonies were therefore more likely to retain remnants following predation. *Porites* and *Montipora* colonies were more frequently left with viable tissue after COTS predation than other genera. In contrast, *Seriatopora* and *Pocillopora* colonies were generally much smaller (see Table 2), with mean colony sizes often below the typical COTS meal size (~96 cm^2^). As a result, smaller colonies were more likely to be consumed entirely, suggesting that the observed differences among genera were largely driven by differences in colony size rather than taxonomic identity. With colony size primarily driving the probability that a remnant is left behind, coral assemblages dominated by larger colonies may be more likely to retain living tissue following COTS predation. If these remnants contribute to colony regeneration, then assemblages dominated by larger colonies may also have greater potential for recovery, whereas those dominated by smaller colonies are less likely to withstand COTS predation.

### 4.3 Evidence for a Consistent Meal Size

Our results indicate that COTS consume a relatively consistent amount of coral tissue during a discrete feeding event, rather than a constant proportion of each colony. This provides a mechanistic explanation for why colony size was the strongest predictor of remnant formation: once colony size exceeds the typical area of tissue consumed in a single feeding event, they are increasingly likely to retain remnants. The scaling relationship between feeding scar area and colony area supports this interpretation. At Second Lagoon, feeding scar area increased less than proportionally with colony area, indicating that larger colonies retained a greater proportion of living tissue following a feeding event. In contrast, feeding scar area scaled approximately in proportion with colony area at Shark Alley. The difference between the sites may reflect differences in the coral community, colony size distributions and/or COTS density, rather than a fundamentally different feeding strategy by COTS itself.

Additional support for a consistent meal‐size comes from feeding events in which live tissue remained despite more than 80% of the colony having been consumed. These events occurred across six coral genera, a wide range of colony sizes and at both study sites. This indicates that incomplete consumption was not restricted to particularly large colonies or specific coral taxa. The remaining tissue in these cases was typically small and is therefore unlikely to represent an unavoidable consequence of colonies being too large to consume completely. Instead, these observations suggest that COTS frequently finish feeding, or move to another coral colony, after consuming a consistent amount of tissue per individual colony, with remnant formation depending primarily on whether the colony exceeds this typical feeding extent.

### 4.4 Evidence That Remnants Show Regenerative Growth Over COTS Feeding Scars

Feeding scars were initially colonised by algal turf, which became denser and more diverse around 10 weeks (~2.5 months) post‐predation, as previously reported (Chesher 1969; Larkum 1988; Belk and Belk 1975). By week 22 (~5 months), CCA began to appear on the scar surface, and in 24% of scars, it fully replaced the initial algal turf. The presence of CCA is ecologically significant, as it promotes coral larval settlement (Abdul Wahab et al. 2023). By week 22, 26% of scars with remnants exhibited at least some regenerative growth of coral over the scar.

Coral tissue regeneration began as early as 5 months post‐predation. Regeneration can be limited by factors such as the size and perimeter of the feeding scar (Meesters and Bak 1997), and the energetic trade‐offs with other vital processes like growth and reproduction (Meesters et al. 1994). Nevertheless, most remnants remained alive at all timepoints monitored, and it is likely that a higher percentage of scars would show signs of regeneration given more time. Future studies could investigate the longer‐term regenerative capacity of feeding scars, i.e., > 1.5 years.

Importantly, all feeding scars monitored over time retained their skeletal structure, showing no evidence of collapse. This aligns with previous findings that reef recovery following COTS outbreaks can be facilitated by the persistence of the reef framework (Colgan 1987). In contrast, coral mortality from severe heat stress and bleaching events can lead to rapid structural collapse and conversion to coral rubble, sometimes within months, as observed for OTR (Byrne et al. 2025). These comparisons suggest that the physical impacts of COTS predation may be less structurally destructive than those associated with ocean warming, potentially allowing for greater recovery potential. Remnants alongside the preserved skeletal structure of the scar, which can provide a stable substrate for new recruits, could enhance reef recovery following COTS predation (Colgan 1987).

### 4.5 Ecological Implications of Remnant Survival

Our findings suggest that individual COTS in a low‐density population feed by sampling multiple colonies rather than consuming individual coral colonies completely. This contrasts with the extensive whole colony mortality characteristic of COTS predation in outbreak populations (Pratchett et al. 2017). The sampling behaviour of COTS at OTI resulted in partial colony predation which left living tissue remnants behind on many corals across all coral genera monitored.

While remnants have been observed previously and hypothesised to be important in tissue regeneration over the scar surface (Done 1985), this has not been previously followed. Of note, some coral genera have tissue that penetrates deep within the skeleton, which have been shown to be an important source of regenerating tissue for wounded coral (Bak and Meesters 1993). Tissue depth within the coral skeleton has been shown to influence regenerative capacity following injury. In *Porites astreoides* , which possesses deep tissue within its perforate skeleton, regeneration was observed to occur from isolated tissue patches within the lesion interior, not just from the lesion margins (Bak and Meesters 1993). In contrast, branching corals typically have shallower tissue layers, which may contribute to their higher mortality following stress events (Loya et al. 2001; Yost et al. 2013). In our study, regenerative growth of tissue over the feeding scar, however, was only observed in coral colonies where visible remnants were left behind, suggesting that regeneration did not occur via other processes (i.e., from deep tissue, larval settlement or tissue overgrowth from adjacent colonies) over the timeframe observed. Although our study tracked only visible surface tissue, future research could explore the role of tissue located deep in the skeleton as a source of regenerative cells following COTS predation.

While all coral genera had evidence of remnants, not all feeding scars showed the capacity to regenerate. *Montipora* and *Porites* had higher proportions of regenerating colonies than other genera. Notably, none of the *Acropora* feeding scars showed signs of regeneration; these remained covered by turf algae or CCA. While previous research has shown that a wide variety of corals can regenerate when live tissue remains (Fishelson 1973), that study did not involve COTS predation. The absence of regeneration in *Acropora* may be due to the nature of COTS damage or insufficient time for recovery. *Seriatopora* and *Pocillopora* were often fully consumed due to their comparatively smaller colony size at OTR, thereby limiting their regenerative capacity following COTS predation.

*Acropora* and *Isopora* (family Acroporidae) are among the fastest growing coral genera (Pratchett et al. 2015), and their removal can promote coral species diversity by reducing competitive dominance and allowing slower‐growing species to persist (Colgan 1987; Done et al. 1997). The feeding behaviour of COTS, leaving higher proportions of remnants in *Montipora* and *Porites* compared to branching corals, with the former also showing a greater ability to regenerate, may contribute to the balance between fast‐ and slow‐growing species on the reef. The sampling feeding behaviour of the OTR population may contribute to increased coral species richness by limiting the dominance of fast‐growing taxa.

**FIGURE 8:**
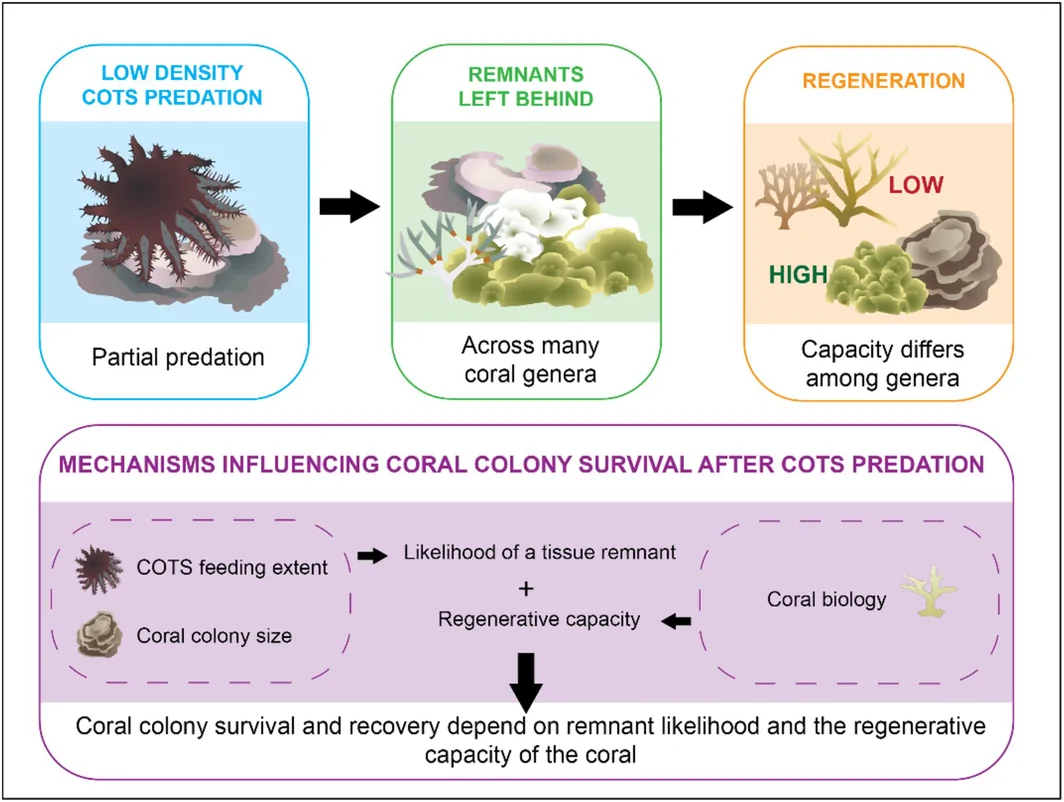
Mechanisms influencing coral colony survival after crown‐of‐thorns‐seastar predation. Symbols courtesy of the Integration and Application Network (ian.umces.edu/media‐library).

Overall, 26% of tracked remnants exhibited regenerative growth, demonstrating that remnants left following COTS predation can remain viable and contribute to partial colony recovery. Thus, for low‐density COTS populations, colonies are not necessarily killed by individual feeding events. Instead, a relatively consistent feeding effort means that larger colonies are more likely to retain living tissue, providing the opportunity for regeneration. The extent of subsequent regeneration differed among coral genera, indicating that colony survival is governed by two interacting processes: the likelihood that a remnant is left following predation, which is primarily determined by COTS feeding behaviour and colony size, and the regenerative capacity of that remnant, which is primarily determined by coral biology (Figure 8). Consequently, COTS feeding may differentially affect coral genera by disproportionately allowing larger, often slower‐growing colonies to survive feeding events and recover over time, potentially contributing to reef resilience under low‐density predation.

### 4.6 Limitations and Future Research

As previously shown, 2D measures of coral colony surface area are linked to 3D volume for corals of the same morphology (e.g., (Urbina‐Barreto et al. 2021)). With the emergence of 3D photogrammetry techniques (Pizarro et al. 2017), we explored whether 3D data would provide additional insights into monitoring changes in the scars. As expected, 3D measured estimated larger scar areas than 2D, but differences were not significant across coral morphologies, suggesting little added value with respect to our specific focus to follow individual scars. However, more accurate estimates of the amount of live coral tissue consumed by COTS would require 3D measurements, particularly to investigate whether the amount of tissue remaining influences the rate of regeneration.

Future research should focus on tracking the long‐term fate of feeding scars alongside changes in coral cover to better understand how COTS predation influences coral diversity and recovery. Tracking the exact fate of individual feeding scars across multiple timepoints would allow an understanding of intrinsic and external factors that influence regeneration of tissue over the scar surface. This will be important for assessing whether all coral genera possess the potential to regenerate following partial predation. Additionally, examining whether COTS revisit previously preyed on colonies following regeneration could provide evidence of sustainable foraging behaviour, whereby COTS facilitate cyclical tissue regrowth and exhibit selective re‐feeding, effectively functioning as ecological gardeners.

## 5 Conclusions

As the first study to track individual feeding scars beyond the initial turf algal colonisation stage, this work extends previous research (Done 1985) by following the longer‐term fate of predation scars. We show that scars retain their skeletal structure and are often colonised by CCA, where living tissue remnants can regenerate over the scars. Regeneration occurred more frequently in slower‐growing genera such as *Montipora* and *Porites*, whereas *Acropora* scars showed no regeneration during the monitoring period. We also found that COTS appear to consume a relatively fixed amount of coral tissue per individual feeding event; consequently, remnant formation is influenced by whether colony size exceeds this typical meal size, rather than by coral genus alone. Consequently, benthic assemblages dominated by larger colonies may have greater potential for remnant‐driven recovery following COTS predation than those dominated by smaller colonies. Together, these findings suggest that, for low‐density populations, COTS exert moderate predation pressure compatible with coral recovery, where viable tissue remnants enable scar regeneration, except in *Acropora*. This selective feeding behaviour may promote coral community diversity by limiting fast‐growing, branching taxa that show a lower regenerative capacity following COTS predation, consistent with the intermediate disturbance hypothesis (Colgan 1987; Done et al. 1997). With establishment of COTS culling programmes in the Great Barrier Reef and through the Indo‐Pacific, increased understanding of low‐density, stable populations is essential to evaluating the functional role of COTS on reefs and the implications of their removal from the ecosystem (Bellwood et al. 2024).

## Author Contributions

**Shawna Andrea Foo:** conceptualization (lead), data curation (lead), formal analysis (lead), funding acquisition (lead), investigation (lead), methodology (lead), resources (lead), supervision (lead), visualization (lead), writing – original draft (lead), writing – review and editing (lead). **Matthew Clements:** investigation (supporting), methodology (supporting). **Adi Zweifler:** formal analysis (equal), methodology (supporting). **Georgie Eggleton:** formal analysis (supporting), investigation (supporting), methodology (supporting). **Hayden Millican:** investigation (supporting), methodology (supporting). **Maria Byrne:** conceptualization (supporting), funding acquisition (supporting), methodology (supporting), writing – review and editing (supporting).

## Funding

This work was supported by Sydney University Horizon Fellowship, Australian Research Council, DE220100555, DP220101125 and Westpac Scholars Trust.

## Conflicts of Interest

The authors declare no conflicts of interest.

## Supporting information


**Table S1:** Metashape Professional workflow followed for construction of three‐dimensional models of crown‐of‐thorns seastar feeding scars.
**Figure S1:** Comparison of 2D (dark grey) versus 3D (light grey) measurements of mean feeding scar size for complex (*n* = 18) and simple (*n* = 11) coral morphology types.
**Table S2:** Results of the linear mixed model analysis investigating the impact of dimension (2D vs. 3D) and coral morphology on feeding scar surface area measurements.
**Table S3:** Results of the logistic regression model investigating the impact of genus and site on whether remnants are left after COTS predation.
**Table S4:** Results of the logistic regression model investigating the impact of genus, site and colony area on whether remnants are left after COTS predation.
**Table S5:** Feeding events where live tissue remained despite > 80% of the coral colony having been consumed.

**Data S1:** COTS_analysis_combined.

**Data S2:** COTS_scar_data.

## Data Availability

Data is available as Supporting Information.
