# Supplementary material for: The Fate of Coral Feeding Scars Following Predation by Individual Crown‐of‐Thorns Seastars
Source: Ecol Evol. 2026 Sep 23;16(9):e74285. doi: 10.1002/ece3.74285 (PMC13600776; doi:10.1002/ece3.74285)
Supplement: Supplementary file 1 [file ECE3-16-e74285-s001.docx]

**Supplementary Information**

**Supplementary Methods**

We trialled the use of three- (3D) and two- (2D) dimensional imaging of the feeding scars to determine if 2D measurements would provide sufficient information on scar size to facilitate efficient field survey with respect to time/tide constraints and shorter data processing time with respect to our aims. In June 2022 and October 2023, alongside the 2D photographs of feeding scars in Shark Alley, additional photographs alongside custom-built scaling objects were captured to create 3D models.

3D models of feeding scars were constructed using Agisoft Metashape Professional version 2.1.0, following procedures outlined by Lange and Perry 2020. Over 100 images were captured and utilised for each different feeding scar (n = 29) to generate the models. The comprehensive workflow for 3D model construction and measurement of 3D surface area is detailed in **Table S1**. 2D surface area was measured in ImageJ (Rasband 2018) by outlining the feeding scar perimeter and subsequently calculating surface area.

We used a linear mixed-effects model with 2D and 3D surface area measurements of the same colonies as the response variable, dimension, coral morphology and their interaction as fixed effects, and colony ID as a random effect. Surface area data were log transformed to meet assumptions of normality and homogeneity, ascertained by visual inspection of model diagnostic plots. Due to low replication at the coral genus level, corals were grouped into morphological categories: “complex” (branching forms) and “simple” (encrusting, massive, and solitary forms).

A comparison of 2D versus 3D measurements of feeding scars in time zero (**Fig. S1**) show that while 3D measurements estimate a significantly larger mean feeding scar size than 2D measurements, this did not differ across complex (branching) or simple (encrusting, solitary, massive) type corals (**Fig. S1**; **Table S2**). It appears that the discrepancy between 2D and 3D estimates (lower and higher, respectively) is not biased by coral growth form. Therefore to facilitate the efficiency of the infield time and post analyses, 2D measurements were used for further comparisons.

**Supplementary Table 1:** **Metashape Professional workflow followed for construction of three-dimensional models of crown-of-thorns seastar feeding scars.**

| Step | Function | Workflow | Description |
| --- | --- | --- | --- |
| 1 | Add Photos | Workflow – Add photos |  |
| 2 | Estimate Image Quality | Reference | Right click on image, estimate image quality and apply to all photos. Check values and disable cameras with values of < 0.3. Remove image if blurry or if field of view does not capture the feeding scar. |
| 3 | Detect Markers | Tools – Markers – Detect Markers | Note that markers refer to scaling objects. |
| 4 | Fix markers | Reference | Right click on each marker. Filter photos by marker. Click on each photo and check that the points are aligned to the correct marker. Where markers are missing, right click and add a marker. If no markers appear, filter through each photo in the photo tab and add new markers or place a marker which already exists. Double check that all markers align with the triangle (shape of the scaling object). |
| 5 | Align Photos | Workflow - Align photos | Accuracy high, no generic or reference preselection, reset current alignment, key point limit 40,000, tie point limit 4,000, apply masks to none and no adaptative camera model fitting. |
| 6 | Build Dense Cloud | Workflow – Build Dense Cloud | Quality high, depth filtering moderate, calculate point colours, and don’t reuse depth maps. |
| 7 | Build Mesh | Workflow – Build Mesh | Source dense cloud, surface type arbitrary, depth map quality medium, face count high, interpolation enabled, point classes all, calculate vertex colours and don’t reuse depth maps or strict volumetric masks. |
| 8 | Build Texture | Workflow – Build Texture | Mapping mode generic, texture from all cameras, mosaic blending mode, texture size 8,192, texture count 1, fill holes and enable ghosting filter. |
| 9 | Scale | Reference | Highlight and right click on two markers which are adjacent (e.g. target 42 + 23), create scale bar. Double click distance and enter 0.06 (6cm). Add as many pairs as possible, refresh the model in the reference tab. |
| 10 | Trim | Catalogue– 3D model | Right click and duplicate the model. Using the Ortho free-form selection tool highlight and delete any living or non-coral substrate. Delete markers once scaled. |
| 11 | Surface Area | Tools – Mesh – Measure area and Volume. |  |

**Supplementary Figure 1. Comparison of 2D (dark grey) versus 3D (light grey) measurements of mean feeding scar size for complex (n = 18) and simple (n = 11) coral morphology types.** Errors bars ± SE.

**Supplementary Table 2. Results of the linear mixed model analysis investigating the impact of dimension (2D vs 3D) and coral morphology on feeding scar surface area measurements.** Significant effects are bolded.

|  | **Estimate** | **Std. error** | **df** | **t-value** | **p** |
| --- | --- | --- | --- | --- | --- |
| **Intercept** | **4.51997** | **0.19810** | **30.21741** | **22.817** | **< 2e-16** |
| **Dimension** | **1.48541** | **0.09411** | **27.00000** | **15.784** | **3.73e-15** |
| Morphology | 0.39094 | 0.32165 | 30.21741 | 1.215 | 0.2336 |
| Dimension x Morphology | -0.26879 | 0.15280 | 27.00000 | -1.759 | 0.0899 |

**Supplementary Table 3. Results of the logistic regression model investigating the impact of genus and site on whether remnants are left after COTS predation.** Significant effects are bolded.

|  | **Estimate** | **Std. error** | **z** | **p** |
| --- | --- | --- | --- | --- |
| **Intercept** | **2.163** | **0.355** | **6.1** | **<0.001** |
| *Isopora* | -0.196 | 0.567 | -0.346 | 0.729 |
| *Montipora* | 0.213 | 0.714 | 0.299 | 0.765 |
| ***Pocillopora*** | **-2.649** | **0.604** | **-4.387** | **<0.001** |
| *Porites* | 0.222 | 0.515 | 0.431 | 0.666 |
| ***Seriatopora*** | **-1.807** | **0.455** | **-3.971** | **<0.001** |
| **Site** | **-1.251** | **0.37** | **-3.383** | **<0.001** |

**Supplementary Table 4. Results of the logistic regression model investigating the impact of genus, site and colony area on whether remnants are left after COTS predation.** Significant effects are bolded.

|  | **Estimate** | **Std. error** | **z** | **p** |
| --- | --- | --- | --- | --- |
| *Isopora* (vs *Acropora*) | 0.696 | 0.848 | 0.821 | 0.412 |
| *Montipora* (vs *Acropora*) | −0.108 | 1.035 | −0.104 | 0.917 |
| *Pocillopora* (vs *Acropora*) | −0.850 | 0.796 | −1.067 | 0.286 |
| *Porites* (vs *Acropora*) | 1.31 | 0.859 | 1.524 | 0.127 |
| *Seriatopora* (vs *Acropora*) | −0.218 | 0.68 | −0.320 | 0.749 |
| Site | −0.412 | 0.472 | −0.872 | 0.383 |
| **Colony area** | **0.756** | **0.264** | **2.863** | **0.004** |

**Supplementary Table 5. Feeding events where live tissue remained despite > 80 % of the coral colony having been consumed.**

| **Genus** | **n** | **Minimum size cm²** | **Maximum size cm²** |
| --- | --- | --- | --- |
| *Seriatopora* | 7 | 25.8 | 142 |
| *Porites* | 4 | 31 | 293 |
| *Isopora* | 3 | 63.9 | 625 |
| *Pocillopora* | 3 | 69.8 | 211 |
| *Acropora* | 2 | 50.8 | 70.9 |
| *Montipora* | 1 | 90.3 | 90.3 |
